# Heterogeneity of White Matter Hyperintensity and Cognitive Impairment in Patients with Acute Lacunar Stroke

**DOI:** 10.3390/brainsci12121674

**Published:** 2022-12-06

**Authors:** Mengfan Ye, Yun Zhou, Huiru Chen, Sijia Zhu, Shanshan Diao, Jieji Zhao, Yan Kong, Tan Li

**Affiliations:** Department of Neurology, The First Affiliated Hospital of Soochow University, No. 899, Pinghai Road, Suzhou 215006, China

**Keywords:** white matter hyperintensity, cognitive impairment, acute lacunar stroke, cerebral small-vessel disease, heterogeneity

## Abstract

Background: The severity of white matter hyperintensity (WMH) in patients with acute lacunar stroke (ALS) may be not completely parallel to cognitive impairment. Controversies persist about the effects of WMH on cognitive dysfunction. It is vital to explore whether the association may be affected by certain factors and whether a subsequent subgroup analysis is necessary. The aim of this study was to evaluate the relationship between WMH and cognitive impairment in acute lacunar stroke patients and the possible causal factors. Methods: We continuously enrolled patients with ALS who were hospitalized at the First Affiliated Hospital of Soochow University between October 2017 and June 2022. The cognitive function of all patients was assessed by using the Montreal Cognitive Assessment (MoCA) scale 14 ± 2 days after the onset of AIS, and the results were adjusted to the education level. The MoCA scale was reevaluated at the 6-month (day 182 ± 7) follow-up by outpatient visit or video. Demographic and clinical data were collected. The manifestations of chronic cerebral small-vessel disease (CSVD), including the total Fazekas score and total CSVD burden score, were assessed with an MRI scan. A mismatch refers to an inconsistency between the severity of WMH and cognitive dysfunction. A Type 1 mismatch refers to cognitive impairment with mild WMH (total Fazekas score = 0–1), and a Type 2 mismatch refers to severe WMH (total Fazekas score = 5–6) in patients with normal cognitive function. Results: Among 213 enrolled ALS patients, 66 patients (31.0%) had cognitive dysfunction, and 40 patients (18.8%) had mismatches. Twenty-seven cases (12.7%) were Type 1 mismatched, and seventeen cases (8.0%) were Type 2 mismatched. Age, gender, fibrinogen and cerebral infarction history were independent risk factors for cognitive impairment in ALS patients. Imaging features, including moderate to severe WMH, deep WMH and the total CSVD burden score, were also independently associated with cognitive impairment. The patients in the mismatched group were older, had more severe deep WMH and had a higher occurrence of depression (*p* < 0.05). The NIHSS score, depression and microbleeds were significantly different between the Type 1 mismatched group and the matched group (*p* = 0.018, *p* = 0.012 and *p* = 0.047). Patients in the Type 2 mismatched group were male (*p* = 0.04), had a lower level of fibrinogen (*p* = 0.005), a lower incidence of CMBs (*p* = 0.003), a lower total CSVD burden score (*p* = 0.017), more severe paraventricular WMH (*p* = 0.035) and milder deep WMH (*p* = 0.026). Conclusions: Our study examined a homogeneous study cohort of recruited patients with symptomatic ALS. We found heterogeneity between WMH and cognitive function in ALS patients. Despite a similar WMH severity, some baseline clinical features and other conventional CSVD imaging characteristics may account for this heterogeneity phenomenon. Our findings provide data for the early diagnosis and prevention of cognitive impairment in ALS patients and suggest that the severity of WMH is not completely parallel to cognitive impairment. The white matter microstructural injury and remote WMH effects may account for the mismatch phenomenon. More attention should be paid to understanding the underlying mechanisms and finding new imaging markers.

## 1. Introduction

Acute lacunar stroke (ALS) accounts for about 25% of all acute ischemic strokes and is one of the most important subtypes of cerebral small-vessel diseases (CSVDs) [1,2]. The imaging manifestation of ALS is a recent small subcortical infarct in the supply area of a small perforating artery [2,3]. Studies have found that 11–23% of patients with lacunar strokes develop persistent cognitive impairment over time, mainly manifested in the impairment of attention and executive function [4]. White matter hyperintensity (WMH) is another important subtype of CSVD and is easily detected in magnetic resonance (MR) examination in elderly people with or without acute ischemic cerebrovascular disease.

In multiple population-based studies, WMHs were reported to be associated with the impairment of cognitive function [5,6,7]. A meta-analysis including thirty-six prospective studies with 19,040 enrolled patients discovered that WMH at baseline was associated with a 14% increased risk of cognitive impairment, and periventricular WMH was related to a 1.51-fold risk of the occurrence of dementia [8]. Cross-sectional studies also suggested that WMHs with higher Fazekas visual grades were correlated with the impairment of specific cognitive domains, but some studies showed weak evidence of effects [9,10,11,12,13,14,15]. Schmidt et al. found that the WMH load was no longer obviously related to cognitive function when adding brain volume changes to the regression model [16]. Moreover, Chen et al. reported a non-linear correlation between WMHs and cognition [17]. Interestingly, different cognitive states have been observed clinically for the same white matter lesions. Therefore, exploring whether the relationship between WMH and cognition is affected by certain factors is crucial, and a further subgroup analysis is warranted. Semi-quantitative visual ratings such as the Fazekas visual grade are often used to assess the severity of WMH. However, there is a ceiling effect in the visual assessment, which does not accurately identify white matter changes [17]. White matter changes are caused by fluid dynamic changes and gradually develop into WMH in the late stage [18]. Studies have found that the progression of WMHs but not baseline white matter was significantly related to cognitive impairment [17,19]. Therefore, more accurate WMH measures are required for better predicting cognitive impairment.

Studies concerning the heterogeneity phenomenon between WMH severity and cognitive impairment have been rare. Wang et al. reported that chronic CSVD patients who had the same degree of WMH suffered different outcomes of cognitive impairment and analyzed the correlated factors [20].

In this study, we aimed to observe the heterogeneity between WMH and cognitive function in ALS patients, identify the vascular risk factors and imaging features that contribute to this heterogeneity and analyze their underlying mechanisms.

## 2. Materials and Methods

### 2.1. Patients

The present study was a prospective observational study with data collected from the neurological department of the First Affiliated Hospital of Soochow University between October 2017 and June 2022. We consecutively recruited patients who were clinically diagnosed with ALS with imaging-proven lacunar infarction lesions. The exclusion criteria were as follows: (1) cortical lesions and/or subcortical lesions larger than 20 mm at the axial diameter on diffusion-weighted imaging (DWI); (2) history of other central nervous system diseases that may lead to WMH, such as encephalitis and multiple sclerosis; (3) severe systemic or other diseases that clearly affect cognition, such as Alzheimer’s disease, hydrocephalus, Parkinson’s disease, etc.; (4) cardioembolic or macroangiopathy (≥50% intracranial or extracranial large-artery atherosclerosis stenosis shown by carotid and cerebrovascular ultrasound, CTA or MRA); (5) history of severe depression (17-item Hamilton Depression Rating Scale score ≥ 24) or other mental illnesses; (6) unable to complete the neuropsychological evaluation due to, for example, disturbance of consciousness or severe language disorders; (7) unable to undergo MR examination. Demographic characteristics, laboratory data and imaging information were collected for all patients. The study was approved by the ethics committee of the First Affiliated Hospital of Soochow University (NO. 2020157), and all participants gave written informed consent.

### 2.2. Data Collection and Baseline Evaluation

Demographic and clinical baseline data were collected within 48 h of admission and included age, gender, smoking and alcohol consumption, past disease history, education level, baseline National Institutes of Health Stroke Scale (NIHSS) score and other relevant laboratory data.

### 2.3. MRI Imaging

All MR imaging was performed on the same 3.0 T MRI scanner (Philips Healthcare, Best, The Netherlands). The magnetic resonance sequences were as follows: T1-weighted, T2-weighted, DWI, T2-FLAIR, 3d-TOF-MRA and susceptibility-weighted imaging (SWI).

### 2.4. CSVD Imaging Markers

The total CSVD burden score was composed of the following four imaging markers. WMH refers to the periventricular and deep white matter T2-weighted MRI areas of high intensity, representing gliosis, axonal loss and ischemic demyelination [21]. One point was assigned for moderate to severe WMH if periventricular WMH was deep (Fazekas score = 3), or deep WMH showed early confluence or confluence (Fazekas score = 2 or 3) [21]. An enlarged perivascular space (EPVS) is generally less than 3 mm, and the shape is linear, round or oval. The signal intensity of EPVS on all MR sequences is similar to that of cerebrospinal fluid (CSF). A number of EVPS > 10 would allocate a 1-point score [9]. A lacune is a round or oval cavity of fluid between 3 mm and 15 mm in diameter under the cortex (signal similar to CSF). Cerebral microbleeds (CMBs) are defined as homogeneous, circular, low-intensity lesions < 5 mm in diameter on SWI. One point was assigned each for the existence of any lacunes or CMBs.

We added up the Fazekas scores of both periventricular WMH and deep WMH to obtain the total WMH Fazekas score. The total Fazekas score (0–6) was used to assess white matter severity, including mild WMH (total Fazekas score = 0–2), moderate WMH (total Fazekas score = 3–4) and severe WMH (total Fazekas score = 5–6). A Type 1 mismatch refers to mild WMH with cognitive impairment, and a Type 2 mismatch refers to severe WMH with normal cognition [20].

### 2.5. Neuropsychological Assessment and Follow-Up Visit

The neuropsychological scale was evaluated by two experienced investigators (Yun Zhou and Shanshan Diao) 14 ± 2 days after stroke onset. The Montreal Cognitive Assessment (MoCA) has high sensitivity and specificity for mild cognitive impairment. According to the Guidelines from the Vascular Impairment of Cognition Classification Consensus Study (VICCCS) [22] Chinese Revision Plan, cognitive impairment is scored as follows: no education, MOCA score ≤ 13; 1–6 years of education, MOCA score ≤ 19; and 7 years of education or more, MOCA score ≤ 24 points. For example, a patient with a MOCA score of 20 is considered cognitively normal if the years of education are less than or equal to six, but this individual is considered to have cognitive impairment if the years of education are seven or more. In addition, the 17-item Hamilton Depression Scale was used for depression severity assessment, and ≤7 points represented a normal condition [23]. We conducted the follow-up visit at 6 months (day 182 ± 7) after the onset of stroke by outpatient visit or video. The items included the neuropsychological scale assessment, mRS score evaluation and medication situation.

### 2.6. Statistical Analysis

The SPSS 26.0 software package was used for statistical analysis. Continuous measurement data conforming to a normal distribution were expressed as mean ± standard deviation (X ± S), measurement data conforming to an abnormal distribution were expressed as median (interquartile range), and count data were expressed as a percentage (%). The independent-sample *t*-test was used for comparisons between two groups of normally distributed measurement data, a non-parametric test was used for measurement data that did not follow a normal distribution, and the chi-square test was used for count data. Variables with *p* < 0.05 in univariable analysis were included in multivariable logistic regression analysis. A *p* value <0.05 was considered statistically significant.

## 3. Results

### 3.1. Baseline Clinical Features and MRI Characteristics

**Figure 1:**
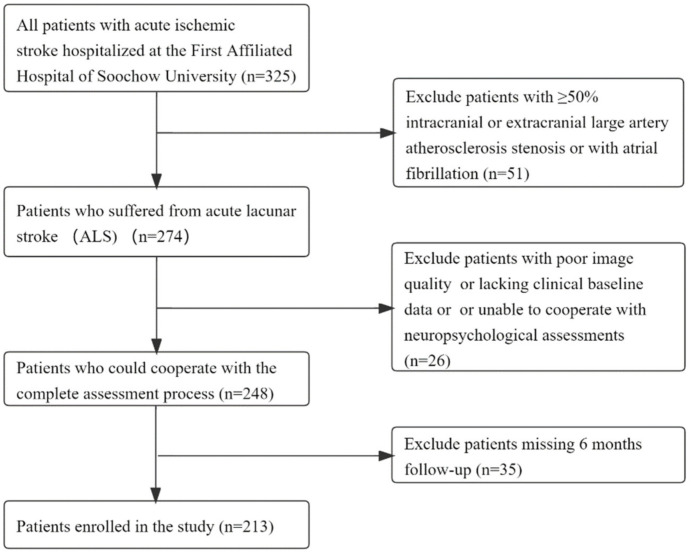
Flow diagram of included and excluded patients.

The flow diagram of patient inclusion and exclusion is shown in Figure 1. After screening all 325 ALS patients admitted, 213 patients were finally enrolled in our study according to the inclusion and exclusion criteria.

**Figure 2:**
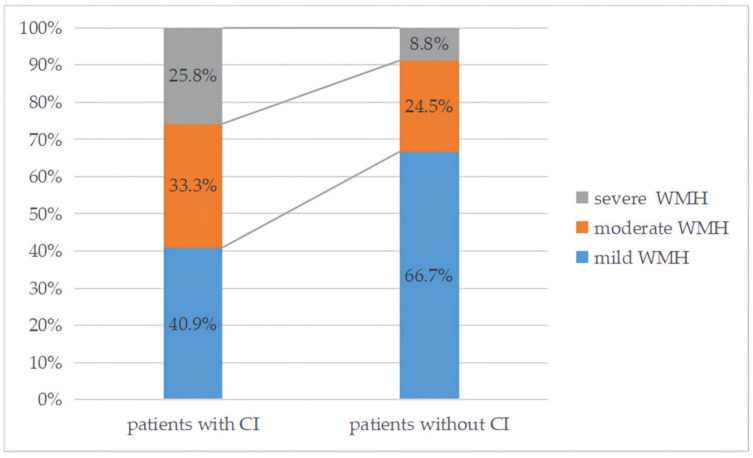
Distribution of WMH severity in patients with and without cognitive impairment. CI, cognitive impairment; mild WMH (Fazekas score = 0–2); moderate WMH (Fazekas score = 3–4); severe WMH (Fazekas score = 5–6).

The clinical and MRI characteristics of the patients at baseline are shown in Table 1. There were 66 patients (31.0%) in the cognitive impairment group with an average age of 68 years (62–76), and 35 were men. The median age was 62 (53–72) years old in the non-cognitive impairment group with 68.7% males. The total CSVD burden scores were 2 (1–3) in the cognitive impairment group and 1 (0–2) in the non-cognitive impairment patients. Moderate to severe WMHs were found in 59.1% of the cognitively impaired ALS patients and 33.3% of the normal group (Figure 2). Univariate analysis showed that the differences between the two groups were age, gender, a previous history of ischemic stroke, the NIHSS scale, hemoglobin, fibrinogen, moderate to severe WMH, lacunes, CMBs, EPVS, deep WMH and the total CSVD burden score (*p* < 0.05).

### 3.2. Independent Risk Factors for Cognitive Impairment in ALS Patients

Multivariable logistic regression analysis showed that age, gender, cerebral infarction history, fibrinogen, moderate to severe WMH and deep WMH were important determinants of long-term cognitive impairment after ALS. A total CSVD burden score of 4 was also one of the independent risk factors of cognitive impairment compared to a score of 0 (Table 2).

### 3.3. Comparison of Clinical Features and CSVD Characteristics between Mismatch and Match Types

**Figure 3:**
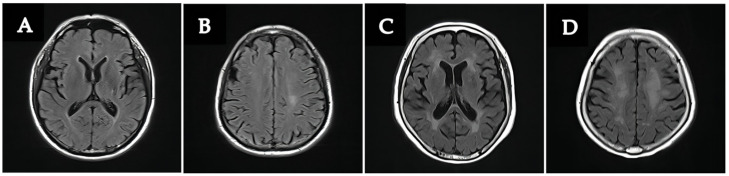
The T2-FLAIR sequence of two typical patients who had heterogeneity between WMH severity and cognition. Case 1: A 51-year-old male patient presented with mild WMHs (**A**,**B**) but had cognitive impairment with MOCA score of 20 (years of education: 12); Case 2: A 60-year-old female patient had normal cognition but severe WMHs (**C**,**D**). The MOCA score was 26 (years of education: 6).

Among all patients, 40 patients (18.8%) had mismatches between WMH severity and cognition, and 115 patients (54.0%) were consistent (Table 3). Figure 3 shows two typical cases of the mismatch types. The patients in the mismatched group were older, had a higher occurrence of depression and had a higher Fazekas score of deep WMH (*p* < 0.05).

Among all of the patients with mismatch types, 27 cases (12.7%) had Type 1 mismatches, and 13 cases (6.1%) had Type 2 mismatches (Table 4). The NIHSS score, depression and CMBs were significantly different between the Type 1 mismatched group and the matched group (*p* = 0.018, *p* = 0.012 and *p* = 0.047). Patients in the Type 2 mismatched group were male (*p* = 0.04), had a lower level of fibrinogen (*p* = 0.005), a lower incidence of CMBs (*p* = 0.003) and a lower total CSVD burden score (*p* = 0.017). We found that both periventricular WMH and deep WMH were associated with a Type 2 mismatch (*p* = 0.035 and *p* = 0.026). In the Type 1 mismatched group, neither periventricular WMH nor deep WMH had any statistical significance (*p* = 0.613 and *p* = 0.560).

## 4. Discussion

This study primarily showed that there was a mismatch between the severity of WMH and cognitive impairment, and this mismatch was associated with some baseline clinical features and other pre-existing CSVD imaging features, such as CMBs, deep WMH and the total CSVD burden. To our best knowledge, this study was the first study that focused on the heterogeneity between WMH and cognitive function in ALS patients.

Previous studies have identified that a variety of vascular risk factors are closely associated with cognitive impairment after stroke, such as hypertension, hyperlipidemia, diabetes mellitus and a previous history of stroke [24,25]. However, few studies have specifically targeted patients with ALS. Our study revealed that higher age, female sex, cerebral infarction history and a higher level of fibrinogen were independently associated with persistent cognitive impairment after lacunar stroke. Yasumasa et al.’s study also indicated that age > 75 years old and female gender were independent risk factors of cognitive impairment in ALS patients [26]. Older age is consistently considered a risk factor for cognitive impairment after stroke. On the one hand, underlying diseases such as hypertension, diabetes mellitus and a history of stroke are common in the older population, which may damage their cognitive function. On the other hand, the susceptibility to cognitive impairment in older people may result from the increased formation of total amyloid beta (Aβ) and Aβ-42 [27,28]. The impact of gender on cognition has not been systematically explored, and our previous study found the female gender to be an independent risk factor of post-stroke cognitive impairment [23]. Several cross-sectional studies have confirmed that elevated fibrinogen was associated with vascular dementia and Alzheimer’s disease [29,30,31,32]. Fibrinogen may reduce blood flow and enhance thrombosis. Meanwhile, fibrinogen is a biomarker of systemic nonspecific inflammation that mediates CAA-related vascular injury [29]. Low et al.’s study found that elevated fibrinogen was associated with the increased appearance of CSVD imaging markers, particularly EVPS and CMBs [29]. Therefore, increased fibrinogen may indicate a poorer cognitive outcome, mainly manifested in decreased executive function and non-verbal reasoning [31]. Our study also revealed that a higher level of fibrinogen was related to ALS patients’ cognitive impairment.

Concerning the relationship between CSVD imaging manifestations and persistent cognitive impairment after ALS, we found that WMH severity, deep WMH and the total CSVD burden were independent risk factors, which are in accordance with previous research [20,33,34,35,36]. However, previous findings have been controversial. For instance, regarding the total CSVD burden score, the results of Wang et al. indicated that the score was independently related to cognition in patients with acute or chronic CSVD [20]. Nevertheless, another study suggested that the total burden score was negatively associated with cognitive impairment in patients suffering from both hypertension and lacunar stroke [37]. Zhi et al. also reported that both the total and modified CSVD scores were not correlated with cognitive function in patients with acute lacunar stroke [38].

Several studies have focused on the association between WMH and cognition, but controversies persist [11,12,39,40,41]. Multiple population-based studies have determined that a larger volume of WMHs, accompanied by higher Fazekas visual grades, is associated with cognitive decline [8,13,15,17]. A meta-analysis reflected that WMHs were closely related to the incidence of cognitive impairment [17]. Specifically, people with WMH had a two-fold increased risk of dementia compared with healthy people, and a higher risk of cognitive impairment was associated with more severe WMH [13]. The LADIS study also found a relationship between WMH and deterioration in several cognitive domains, including clinical memory, attention, executive function and overall cognition [41]. However, a review reported that cognitive function in patients with the same WMH volume or visual rating score could be either impaired or preserved [17], which was also observed in our clinical exercise. Previous studies have found heterogeneous performance in WMH [20,42,43]. Wardlaw et al. discovered that the white matter lesion volume might increase or decrease after cerebral infarction, and controlling risk factors could reverse WMH-related brain damage [44]. Studies concerning the heterogeneity between WMH severity and cognitive impairment have been rare. Yilong Wang et al. reported that heterogeneity existed between WMH severity and cognitive impairment in CSVD patients, with 23.7% of the patients presenting mismatches [20]. As far as we know, this study was the first study that focused on the heterogeneity between WMH and cognitive function in ALS patients, and we found a mismatch existed with a proportion of 18.8%. This mismatch was associated with age, depression and deep WMH. We further distinguished mismatches of Type 1 and Type 2. We found that the Type 1 mismatched group had a higher NIHSS score, a higher rate of depression and a higher incidence of CMBs (*p* = 0.018, *p* = 0.012 and *p* = 0.047). Patients in the Type 2 mismatched group were male (*p* = 0.04), had a lower level of fibrinogen (*p* = 0.005), a lower incidence of CMBs (*p* = 0.003), a lower total CSVD burden score (*p* = 0.017), more severe paraventricular WMH (*p* = 0.035) and less severe deep WMH (*p* = 0.026). The NIHSS score could reflect the severity of the stroke event [45]. The study of Einstad et al. also suggested that a higher NIHSS score may increase the risk of cognitive impairment [46]. Post-stroke depression occurs in approximately 31% of patients with acute ischemic stroke [47]. Depression was related to neurobiological dysfunction due to ischemic injury and psychosocial factors [48,49]. Several studies also indicated that patients with depression suffered more severe cognitive impairment than those without depression, which is in agreement with our findings [49,50,51]. The results of Wardlaw et al. indicated that both periventricular WMH and deep WMH were related to cognition in elderly people [2]. De Groot et al. reported that severe periventricular WMH could predict the dynamic change in cognition in elderly adults [52], while deep WMH was associated with depressive symptoms [53]. In our results, deep WMH rather than periventricular WMH was found to be associated with cognitive impairment and the mismatch between WMH and cognitive function in ALS patients. WMH may affect cognition through the following mechanisms. Firstly, white matter lesions may directly damage subcortical neural networks [54,55]. Secondly, WMH as a confounder reflects the association between vascular risk factors and cognitive impairment [56]. Finally, WMHs were shown to be associated with dementia-related pathological processes, such as cerebral amyloid angiopathy and the lack of myelin occurring secondary to neuronal loss [8,21,56].

Two mechanisms probably account for inconsistent clinical presentations. Firstly, focal WMH lesions may progressively impair the surrounding normal tissues and damage the white matter integrity, which could cause damage to both anatomical structures and functional networks [17]. One study suggested that the original focal lesion might lead to abnormal dispersion, even over 200 times the original damage volume [57]. Recently, the concept of normal-appearing white matter (NAWM) was proposed, which refers to white matter near the WMH lesion that is observed to be normal on conventional MRI sequences but has damage to its microstructure [58,59,60,61,62]. Some studies reported that NAWM was related to cognitive impairment [58,59]. Thus, a change in the microstructure of white matter may in part explain the mismatch phenomenon. Secondly, focal lesions caused by WMHs can spread to remote areas through a number of pathophysiological changes [63]. In addition, studies found that WMHs and hippocampal atrophy had a cumulative effect, and periventricular WMHs were connected to hippocampal atrophy [15,17,35,44]. Taken together, the heterogeneity between WMH and cognition may be attributed to the white matter microstructural injury and remote WMH effects.

The presence of WMH on the conventional MRI sequence indicates the injury of cerebral small vessels. However, the subtypes of CSVD interact with each other. For instance, one study showed that the margin of the white matter lesion was usually the preferred site for lacunar occurrence [64]. Another study showed that CSVD patients had less reduction in white-matter free water around EPVS than healthy subjects [65]. Moreover, the coexistence of various subtypes of CSVD in one patient is common. Thus, the heterogeneity between WMH and cognitive impairment in our study can be partially explained. Nowadays, more and more evidence is indicating that CSVD should be considered a global cerebral disease, which implies that focal lesions affect distal brain structures and structural and functional network connectivity [63]. The conventional MRI sequence is far from enough to evaluate the influence of CSVD on the brain, and new imaging technology and imaging signs should be explored in the future.

In our study, we focused on the mismatch between white matter severity and cognitive impairment, defined as Type 1 and Type 2 mismatches, and conducted a subgroup analysis. The main advantage was the homogeneous study cohort of the recruited patients with symptomatic ALS. Moreover, our longitudinal design allowed an assessment for long-term cognitive impairment.

However, our study also had several limitations. First, this study was monocentric with a small sample, and the sampling bias and possible selection bias were considered. Second, although the MOCA scale has good specificity and sensitivity, more detailed scales should be carried out for cognitive domain assessment. Third, several patients were excluded because of aphasia or the disturbance of consciousness, and yet these patients may suffer from more severe strokes. Therefore, the patients included in our study may have had a relatively mild stroke attack. Finally, the volume of WMH was not calculated, and the location of WMH was not recorded in this study, which might be associated with cognitive function.

## 5. Conclusions

The severity of WMH was not completely parallel to cognitive impairment. Despite a similar WMH severity, differences in some baseline clinical features and other conventional CSVD imaging characteristics may result in different cognitive outcomes. Our findings suggest nonconformity exists between WMH severity and cognitive impairment in ALS patients and indicates that new imaging markers should be explored.

## Figures and Tables

**Table 1:** Demographic, clinical and CSVD characteristics of patients.

	Total Patients (n = 213)	With Cognitive Impairment (n = 66)	Without Cognitive Impairment (n = 147)	*p* Value	BH-Adjusted *p* Values (*q* Value)
Demographic and clinical characteristics					
Age (years)	64 (56–72)	68 (62–76)	62 (53–72)	*p* = 0.001 *	*q* = 0.004 ^#^
Male, n (%)	136 (63.8%)	35 (53.0%)	101 (68.7%)	*p* = 0.028 *	*q* = 0.061 ^#^
Medical history					
Cerebral infarction, n (%)	46 (21.6%)	21 (31.8%)	25 (17.0%)	*p* = 0.015 *	*q* = 0.043 ^#^
Hypertension, n (%)	159 (74.6%)	53 (80.3%)	106 (72.1%)	*p* = 0.204	*q* = 0.295
Diabetes mellitus, n (%)	59 (27.7%)	22 (33.3%)	37 (25.2%)	*p* = 0.218	*q* = 0.298
MOCA score	22 (19–25)	20 (16–24)	24 (23–26)	*p* < 0.001 *	*q* = 0.001 ^#^
Depression, n (%)	18 (8.5%)	8 (12.1%)	10 (6.8%)	*p* = 0.197	*q* = 0.295
NIHSS score	3 (1–3)	4 (1–5)	2(1–3)	*p* = 0.018 *	*q* = 0.043 ^#^
Laboratory examination					
Thrombocyte (10^9^/L)	201 ± 58	201 ± 60	202 ± 59	*p* = 0.793	*q* = 0.825
LDL-C (mmol/L)	2.64 (2.07–3.14)	2.63 (2.08–3.05)	2.65 (2.07–3.16)	*p* = 0.360	*q* = 0.446
Hemoglobin (g/L)	134 (124–144)	131 (121–142)	135 (126–146)	*p* = 0.035 *	*q* = 0.070 ^#^
Creatinine (μmol/L)	71 (57–80)	70 (60–81)	71 (57–80)	*p* = 0.685	*q* = 0.759
hs-CRP (mg/L)	3.75 (0.76–4.71)	4.21 (0.85–4.97)	3.59 (0.66–4.59)	*p* = 0.136	*q* = 0.236
Fibrinogen (g/L)	2.72 (2.14–3.13)	1.75 (2.37–3.37)	1.64 (2.00–3.04)	*p* = 0.001 *	*q* = 0.004 ^#^
Imaging features					
Infarction lesions					
Thalamus	25 (11.7%)	8 (12.1%)	17 (11.6%)	*p* = 0.907	*q* = 0.907
Basal ganglia/internal capsule	82 (38.5)	27 (40.9%)	55 (37.4%)	*p* = 0.628	*q* = 0.742
Centrum ovale/corona radiata	70 (32.9%)	18 (27.3%)	52 (35.4%)	*p* = 0.244	*q* = 0.317
Medulla/midbrain/pons/cerebellum	52 (24.4%)	15 (22.7%)	37 (25.2%)	*p* = 0.701	*q* = 0.759
WMH Fazekas score (0–6)	2 (0–4)	3 (1–4)	2 (0–3)	*p* = 0.067	*q* = 0.124
Lacune, n (%)	114 (53.5%)	44 (66.7%)	70 (47.6%)	*p* = 0.011 *	*q* = 0.036 ^#^
Microbleeds, n (%)	52 (24.4%)	28 (42.4%)	24 (16.3%)	*p* < 0.001 *	*q* = 0.001 ^#^
EPVS (N > 10), n (%)	109 (51.2%)	42 (63.6%)	67 (45.6%)	*p* = 0.017 *	*q* = 0.043 ^#^
Moderate to severe WMH, n (%)	76 (35.7%)	37 (56.1%)	39 (26.5%)	*p* < 0.001 *	*q* = 0.001 ^#^
Periventricular WMH	1 (0–2)	2 (1–3)	1 (0–2)	*p* = 0.189	*q* = 0.295
Deep WMH	1 (0–1)	1 (0–2)	0 (0–1)	*p* < 0.001 *	*q* = 0.001 ^#^
Total CSVD score (0–4)	2 (1–3)	2 (1–3)	1 (0–2)	*p* = 0.001 *	*q* = 0.004 ^#^

Results are expressed as number (column %), mean ± SD or median (interquartile range). Abbreviations: MOCA, Montreal Cognitive Assessment; NIHSS, National Institutes of Health Stroke Scale; LDL-C, low-density lipoprotein cholesterol; hs-CRP, high-sensitivity C-reactive protein; EPVS, enlarged perivascular space; WMH, white matter hyperintensities; CSVD, cerebral small-vessel disease. * *p* < 0.05. # *q* < 0.1.

**Table 2:** Multivariable logistic regression analysis of risk factors associated with cognitive impairment.

	OR	95% CI	B	*p* Value
Age(years)	1.044	1.009–1.080	0.043	*p* = 0.014 *
Male, n (%)	0.379	0.176–0.817	−0.970	*p* = 0.013 *
NIHSS score	1.124	0.995–1.268	0.116	*p* = 0.060
Cerebral infarction history, n (%)	2.359	1.027–5.419	0.858	*p* = 0.043 *
Fibrinogen (g/L)	1.810	1.242–2.639	0.594	*p* = 0.002 *
Moderate to severe WMH	3.485	1.656–7.333	1.248	*p* = 0.001 *
Deep WMH	6.037	2.600–14.020	1.798	*p* < 0.001 *
Total CSVD score (0–4)				*p* = 0.005 *
0	Ref.	Ref.	Ref.	Ref.
1	0.558	0.189–1.654	−0.583	*p* = 0.293
2	1.116	0.388–3.211	0.110	*p* = 0.839
3	1.878	0.598–5.898	0.630	*p* = 0.281
4	7.309	1.872–28.530	1.989	*p* = 0.004 *

CI, confidence interval; B, regression coefficient; NIHSS, National Institutes of Health Stroke Scale; WMH, white matter hyperintensities; CSVD, cerebral small-vessel disease. * *p* < 0.05.

**Table 3:** Comparison between mismatch and match types.

	Mismatch Type (n = 40)	Match Type (n = 115)	*p* Value
Demographic and clinical characteristics			
Age (years)	67 ± 10	61 ± 12	*p* = 0.012 *
Male, n (%)	25(62.5%)	73 (63.5%)	*p* = 0.912
Depression, n (%)	7(17.5%)	7 (6.1%)	*p* = 0.030 *
Medical history			
Cerebral infarction, n (%)	12(30.0%)	22 (19.1%)	*p* = 0.152
Hypertension, n (%)	31(77.5%)	83 (72.2%)	*p* = 0.511
Diabetes mellitus, n (%)	13(32.5%)	31 (27.0%)	*p* = 0.503
Laboratory examination			
Thrombocyte (10^9^/L)	194 ± 45	204 ± 63	*p* = 0.285
LDL-C (mmol/L)	2.59 (1.91–3.11)	2.65 (2.08–3.10)	*p* = 0.343
Hemoglobin (g/L)	135 (125–145)	134 (125–145)	*p* = 0.864
Creatinine (μmol/L)	72 (59–81)	71 (56–79)	*p* = 0.193
hs-CRP (mg/L)	3.70 (0.79–4.92)	3.77 (0.77–4.47)	*p* = 0.852
Fibrinogen (g/L)	2.55 (2.09–2.91)	2.70 (2.15–3.06)	*p* = 0.422
Imaging features			
Periventricular WMH	1 (0–3)	1 (0–1)	*p* = 0.109
Deep WMH	1 (0–2)	1 (0–1)	*p* = 0.038 *
Lacune, n (%)	22 (55.0%)	59 (51.3%)	*p* = 0.724
Microbleeds, n (%)	10 (25.0%)	25 (21.7%)	*p* = 0.690
EPVS (N > 10), n (%)	22 (55.0%)	49 (42.6%)	*p* = 0.190
Total CSVD score (0–4)	2 (1–3)	1 (0–2)	*p* = 0.082

Mismatch type: cognitively normal but with severe white matter hyperintensity (Fazekas score = 5–6), or cognitively impaired but with mild white matter hyperintensity (Fazekas score = 0–2). Otherwise, we call it a match. LDL-C, low-density lipoprotein cholesterol; hs-CRP, high-sensitivity C-reactive protein; EPVS, enlarged perivascular space; CSVD, cerebral small-vessel disease. WMH, white matter hyperintensity. * *p* < 0.05.

**Table 4:** Features related to Type 1 mismatch and Type 2 mismatch.

	Type 1			Type 2		
	Mismatch (n = 27)	Match (n = 98)	*p* Value	Mismatch (n = 13)	Match (n = 17)	*p* Value
Age (years)	64 (55–73)	59 (51–68)	*p* = 0.081	73 ± 7	73 ± 9	*p* = 0.971
Male, n (%)	13 (48.2%)	63 (64.3%)	*p* = 0.128	12 (92.3%)	10 (58.8%)	*p* = 0.040 *
NIHSS score	4 (1–4)	2 (1–3)	*p* = 0.018 *	3 (0–3)	2 (0–4)	*p* = 0.915
Depression, n (%)	6 (22.2%)	6 (6.1%)	*p* = 0.012 *	1 (7.7%)	1 (5.9%)	*p* = 0.844
Medical history						
Cerebral infarction, n (%)	8 (29.6%)	17 (17.4%)	*p* = 0.158	4 (30.8%)	5 (29.4%)	*p* = 0.936
Hypertension, n (%)	20 (74.1%)	69 (70.4%)	*p* = 0.710	11 (84.6%)	14 (82.4%)	*p* = 0.869
Diabetes mellitus, n (%)	11 (40.7%)	25 (25.5%)	*p* = 0.122	2 (15.4%)	6 (35.3%)	*p* = 0.222
Laboratory examination						
Thrombocyte (10^9^/L)	195 ± 48	204 ± 63	*p* = 0.275	191 ± 38	203 ± 66	*p* = 0.572
LDL-C (mmol/L)	2.62 ± 1.04	2.64 ± 0.77	*p* = 0.091	2.54 (2.28–3.12)	2.71 (2.37–3.10)	*p* = 0.967
Hemoglobin (g/L)	134 (124–143)	135 (126–146)	*p* = 0.477	136 ± 13	129 ± 15	*p* = 0.184
Creatinine (μmol/L)	69 (57–78)	71 (56–78)	*p* = 0.732	78 ± 13	72 ± 16	*p* = 0.287
hs-CRP (mg/L)	3.42 (0.79–3.57)	3.67 (0.67–4.43)	*p* = 0.859	4.26 (0.75–7.23)	4.36 (0.92–4.97)	*p* = 0.706
Fibrinogen (g/L)	2.85 (2.27–3.14)	2.67 (2.08–3.06)	*p* = 0.339	1.93 (1.36–2.45)	2.89 (2.48–3.11)	*p* = 0.005 *
Imaging features						
Periventricular WMH	1 (0–1)	0 (0–1)	*p* = 0.613	3 (3–3)	3 (2–3)	*p* = 0.035 *
Deep WMH	0 (0–0)	0 (0–0)	*p* = 0.560	2 (2–3)	3 (2–3)	*p* = 0.026 *
Lacune, n (%)	15 (55.6%)	45 (45.9%)	*p* = 0.399	7 (53.8%)	14 (82.4%)	*p* = 0.091
Microbleeds, n (%)	8 (29.6%)	13 (13.3%)	*p* = 0.047 *	2 (15.4%)	12 (70.6%)	*p* = 0.003 *
EPVS (N > 10), n (%)	13 (48.1%)	36 (36.7%)	*p* = 0.300	9 (69.2%)	13 (76.5%)	*p* = 0.657
Total CSVD score	1 (0–2)	1 (0–2)	*p* = 0.184	2 (2–3)	3 (3–4)	*p* = 0.017 *

Type 1 mismatch: mild white matter hyperintensity (Fazekas score 0–2) with cognitive impairment; Type 2 mismatch: severe white matter hyperintensity (Fazekas score 5–6) with normal cognition. NIHSS, National Institutes of Health Stroke Scale; LDL-C, low-density lipoprotein cholesterol; hs-CRP, high-sensitivity C-reactive protein. EPVS, enlarged perivascular space; CSVD, cerebral small-vessel disease. WMH, white matter hyperintensities. * *p* < 0.05.

## Data Availability

The datasets generated during the current study are available from the corresponding author on reasonable request.

## Abbreviations

White matter hyperintensity (WMH), acute lacunar stroke (ALS), Montreal Cognitive Assessment (MoCA), cerebral small-vessel disease (CSVD), magnetic resonance (MR), diffusion-weighted imaging (DWI), National Institutes of Health Stroke Scale (NIHSS), susceptibility-weighted imaging (SWI), enlarged perivascular space (EPVS), cerebrospinal fluid (CSF), cerebral microbleed (CMB), Vascular Impairment of Cognition Classification Consensus Study (VICCCS), low-density lipoprotein (LDL-C); high-sensitivity C-reactive protein (hs-CRP); normal-appearing white matter (NAWM).

## References

[1] Jiang S., Wu S., Zhang S., Wu B. (2021). Advances in Understanding the Pathogenesis of Lacunar Stroke: From Pathology and Pathophysiology to Neuroimaging. Cerebrovasc. Dis..

[2] Wardlaw J.M., Smith E.E., Biessels G.J., Cordonnier C., Fazekas F., Frayne R., Lindley R.I., O’Brien J.T., Barkhof F., Benavente O.R. (2013). Neuroimaging standards for research into small vessel disease and its contribution to ageing and neurodegeneration. Lancet Neurol..

[3] Wardlaw J.M., Smith C., Dichgans M. (2013). Mechanisms of sporadic cerebral small vessel disease: Insights from neuroimaging. Lancet Neurol..

[4] Edwards J.D., Jacova C., Sepehry A.A., Pratt B., Benavente O.R. (2013). A quantitative systematic review of domain-specific cognitive impairment in lacunar stroke. Neurology.

[5] De Groot J.C., de Leeuw F.E., Oudkerk M., van Gijn J., Hofman A., Jolles J., Breteler M.M. (2000). Cerebral white matter lesions and cognitive function: The Rotterdam Scan Study. Ann Neurol..

[6] Inzitari D., Pracucci G., Poggesi A., Carlucci G., Barkhof F., Chabriat H., Erkinjuntti T., Fazekas F., Ferro J.M., Hennerici M. (2009). Changes in white matter as determinant of global functional decline in older independent outpatients: Three year follow-up of LADIS (leukoaraiosis and disability) study cohort. BMJ.

[7] Wang Y.L., Chen W., Cai W.J., Hu H., Xu W., Wang Z.T., Cao X.P., Tan L., Yu J.T. (2020). Associations of White Matter Hyperintensities with Cognitive Decline: A Longitudinal Study. J. Alzheimer’s Dis..

[8] Hu H.Y., Ou Y.N., Shen X.N., Qu Y., Ma Y.H., Wang Z.T., Dong Q., Tan L., Yu J.T. (2021). White matter hyperintensities and risks of cognitive impairment and dementia: A systematic review and meta-analysis of 36 prospective studies. Neurosci. Biobehav. Rev..

[9] Garde E., Mortensen E.L., Krabbe K., Rostrup E., Larsson H.B. (2000). Relation between age-related decline in intelligence and cerebral white-matter hyperintensities in healthy octogenarians: A longitudinal study. Lancet.

[10] Fruhwirth V., Enzinger C., Fandler-Hofler S., Kneihsl M., Eppinger S., Ropele S., Schmidt R., Gattringer T., Pinter D. (2021). Baseline white matter hyperintensities affect the course of cognitive function after small vessel disease-related stroke: A prospective observational study. Eur. J. Neurol..

[11] Silbert L.C., Howieson D.B., Dodge H., Kaye J.A. (2009). Cognitive impairment risk: White matter hyperintensity progression matters. Neurology.

[12] Debette S., Bombois S., Bruandet A., Delbeuck X., Lepoittevin S., Delmaire C., Leys D., Pasquier F. (2007). Subcortical hyperintensities are associated with cognitive decline in patients with mild cognitive impairment. Stroke.

[13] Debette S., Markus H.S. (2010). The clinical importance of white matter hyperintensities on brain magnetic resonance imaging: Systematic review and meta-analysis. BMJ.

[14] Kloppenborg R.P., Nederkoorn P.J., Geerlings M.I., van den Berg E. (2014). Presence and progression of white matter hyperintensities and cognition: A meta-analysis. Neurology.

[15] Sivakumar L., Riaz P., Kate M., Jeerakathil T., Beaulieu C., Buck B., Camicioli R., Butcher K. (2017). White matter hyperintensity volume predicts persistent cognitive impairment in transient ischemic attack and minor stroke. Int. J. Stroke.

[16] Schmidt R., Ropele S., Enzinger C., Petrovic K., Smith S., Schmidt H., Matthews P.M., Fazekas F. (2005). White matter lesion progression, brain atrophy, and cognitive decline: The Austrian stroke prevention study. Ann. Neurol..

[17] Chen Y., Wang X., Guan L., Wang Y. (2021). Role of White Matter Hyperintensities and Related Risk Factors in Vascular Cognitive Impairment: A Review. Biomolecules.

[18] De Groot M., Verhaaren B.F., de Boer R., Klein S., Hofman A., van der Lugt A., Ikram M.A., Niessen W.J., Vernooij M.W. (2013). Changes in normal-appearing white matter precede development of white matter lesions. Stroke.

[19] Schmidt R., Berghold A., Jokinen H., Gouw A.A., van der Flier W.M., Barkhof F., Scheltens P., Petrovic K., Madureira S., Verdelho A. (2012). White matter lesion progression in LADIS: Frequency, clinical effects, and sample size calculations. Stroke.

[20] Wang T., Jin A., Fu Y., Zhang Z., Li S., Wang D., Wang Y. (2021). Heterogeneity of White Matter Hyperintensities in Cognitively Impaired Patients With Cerebral Small Vessel Disease. Front. Immunol..

[21] Debette S., Schilling S., Duperron M.G., Larsson S.C., Markus H.S. (2019). Clinical Significance of Magnetic Resonance Imaging Markers of Vascular Brain Injury: A Systematic Review and Meta-analysis. JAMA Neurol..

[22] Skrobot O.A., Black S.E., Chen C., DeCarli C., Erkinjuntti T., Ford G.A., Kalaria R.N., O’Brien J., Pantoni L., Pasquier F. (2018). Progress toward standardized diagnosis of vascular cognitive impairment: Guidelines from the Vascular Impairment of Cognition Classification Consensus Study. Alzheimer’s Dement..

[23] Tang A., Liu S., Wang Z., Shao H., Cai X., Li T. (2022). A New Nomogram Model for Individualized Prediction of Cognitive Impairment in Patients with Acute Ischemic Stroke. J. Stroke Cerebrovasc. Dis..

[24] Pendlebury S.T., Rothwell P.M. (2009). Prevalence, incidence, and factors associated with pre-stroke and post-stroke dementia: A systematic review and meta-analysis. Lancet Neurol..

[25] Surawan J., Areemit S., Tiamkao S., Sirithanawuthichai T., Saensak S. (2017). Risk factors associated with post-stroke dementia: A systematic review and meta-analysis. Neurol. Int..

[26] Yamamoto Y., Ohara T., Nagakane Y., Tanaka E., Morii F., Koizumi T., Akiguchi I. (2011). Chronic kidney disease, 24-h blood pressure and small vessel diseases are independently associated with cognitive impairment in lacunar infarct patients. Hypertens. Res..

[27] Akinyemi R.O., Allan L.M., Oakley A., Kalaria R.N. (2017). Hippocampal Neurodegenerative Pathology in Post-stroke Dementia Compared to Other Dementias and Aging Controls. Front. Neurosci..

[28] Sperling R.A., Aisen P.S., Beckett L.A., Bennett D.A., Craft S., Fagan A.M., Iwatsubo T., Jack C.R., Kaye J., Montine T.J. (2011). Toward defining the preclinical stages of Alzheimer’s disease: Recommendations from the National Institute on Aging-Alzheimer’s Association workgroups on diagnostic guidelines for Alzheimer’s disease. Alzheimer’s Dement..

[29] Low A., Mak E., Rowe J.B., Markus H.S., O’Brien J.T. (2019). Inflammation and cerebral small vessel disease: A systematic review. Ageing Res. Rev..

[30] Van Oijen M., Witteman J.C., Hofman A., Koudstaal P.J., Breteler M.M. (2005). Fibrinogen is associated with an increased risk of Alzheimer disease and vascular dementia. Stroke.

[31] Marioni R.E., Stewart M.C., Murray G.D., Deary I.J., Fowkes F.G., Lowe G.D., Rumley A., Price J.F. (2009). Peripheral levels of fibrinogen, C-reactive protein, and plasma viscosity predict future cognitive decline in individuals without dementia. Psychosom. Med..

[32] Rafnsson S., Deary I.J., Whiteman M.C., Rumley A., Lowe G.D., Fowkes F.G. (2010). Haemorheological predictors of cognitive decline: The Edinburgh Artery Study. Age Ageing..

[33] Nakamori M., Hosomi N., Tachiyama K., Kamimura T., Matsushima H., Hayashi Y., Imamura E., Wakabayashi S., Maruyama H. (2020). Lobar microbleeds are associated with cognitive impairment in patients with lacunar infarction. Sci. Rep..

[34] Yakushiji Y., Noguchi T., Hara M., Nishihara M., Eriguchi M., Nanri Y., Nishiyama M., Hirotsu T., Nakajima J., Kuroda Y. (2012). Distributional impact of brain microbleeds on global cognitive function in adults without neurological disorder. Stroke.

[35] Firbank M.J., Burton E.J., Barber R., Stephens S., Kenny R.A., Ballard C., Kalaria R.N., O’Brien J.T. (2007). Medial temporal atrophy rather than white matter hyperintensities predict cognitive decline in stroke survivors. Neurobiol. Aging.

[36] Van der Flier W.M., van Buchem M.A., van Buchem H.A. (2003). Volumetric MRI predicts rate of cognitive decline related to AD and cerebrovascular disease. Neurology.

[37] Huijts M., Duits A., van Oostenbrugge R.J., Kroon A.A., de Leeuw P.W., Staals J. (2013). Accumulation of MRI Markers of Cerebral Small Vessel Disease is Associated with Decreased Cognitive Function. A Study in First-Ever Lacunar Stroke and Hypertensive Patients. Front. Aging Neurosci..

[38] Zhi N., Zhang L., Wang Y., Bai S., Geng J., Yu L., Cao W., Zhuang L., Zhou Y., Guan Y. (2021). Modified cerebral small vessel disease score is associated with vascular cognitive impairment after lacunar stroke. Aging.

[39] Smith E.E., Blacker D., Killiany R.J., Muzikansky A., Dickerson B.C., Tanzi R.E., Albert M.S., Greenberg S.M., Guttmann C.R. (2008). Magnetic resonance imaging white matter hyperintensities and brain volume in the prediction of mild cognitive impairment and dementia. Arch. Neurol..

[40] Dufouil C., Godin O., Chalmers J., Coskun O., MacMahon S., Tzourio-Mazoyer N., Bousser M.G., Anderson C., Mazoyer B., Tzourio C. (2009). Severe cerebral white matter hyperintensities predict severe cognitive decline in patients with cerebrovascular disease history. Stroke.

[41] Jokinen H., Kalska H., Ylikoski R., Madureira S., Verdelho A., van der Flier W.M., Scheltens P., Barkhof F., Visser M.C., Fazekas F. (2009). Longitudinal cognitive decline in subcortical ischemic vascular disease--the LADIS Study. Cerebrovasc. Dis..

[42] Gouw A.A., Seewann A., van der Flier W.M., Barkhof F., Rozemuller A.M., Scheltens P., Geurts J.J. (2011). Heterogeneity of small vessel disease: A systematic review of MRI and histopathology correlations. J. Neurol. Neurosurg. Psychiatry.

[43] Jung K.H., Stephens K.A., Yochim K.M., Riphagen J.M., Kim C.M., Buckner R.L., Salat D.H. (2021). Heterogeneity of Cerebral White Matter Lesions and Clinical Correlates in Older Adults. Stroke.

[44] Wardlaw J.M., Chappell F.M., Valdes Hernandez M.D.C., Makin S.D.J., Staals J., Shuler K., Thrippleton M.J., Armitage P.A., Munoz-Maniega S., Heye A.K. (2017). White matter hyperintensity reduction and outcomes after minor stroke. Neurology.

[45] Lyden P. (2017). Using the National Institutes of Health Stroke Scale: A Cautionary Tale. Stroke.

[46] Einstad M.S., Saltvedt I., Lydersen S., Ursin M.H., Munthe-Kaas R., Ihle-Hansen H., Knapskog A.B., Askim T., Beyer M.K., Naess H. (2021). Associations between post-stroke motor and cognitive function: A cross-sectional study. BMC Geriatr..

[47] Hackett M.L., Pickles K. (2014). Part I: Frequency of depression after stroke: An updated systematic review and meta-analysis of observational studies. Int. J. Stroke.

[48] Villa R.F., Ferrari F., Moretti A. (2018). Post-stroke depression: Mechanisms and pharmacological treatment. Pharmacol. Ther..

[49] Nys G.M., van Zandvoort M.J., van der Worp H.B., de Haan E.H., de Kort P.L., Kappelle L.J. (2005). Early depressive symptoms after stroke: Neuropsychological correlates and lesion characteristics. J. Neurol. Sci..

[50] Miller M.D., Lenze E.J., Dew M.A., Whyte E., Weber E., Begley A.E., Reynolds C.F. (2002). Effect of cerebrovascular risk factors on depression treatment outcome in later life. Am. J. Geriatr. Psychiatry.

[51] Moorhouse P., Song X., Rockwood K., Black S., Kertesz A., Gauthier S., Feldman H. (2010). Executive dysfunction in vascular cognitive impairment in the consortium to investigate vascular impairment of cognition study. J. Neurol. Sci..

[52] De Groot J.C., De Leeuw F.E., Oudkerk M., Van Gijn J., Hofman A., Jolles J., Breteler M.M. (2002). Periventricular cerebral white matter lesions predict rate of cognitive decline. Ann. Neurol..

[53] De Groot J.C., de Leeuw F.E., Oudkerk M., Hofman A., Jolles J., Breteler M.M. (2000). Cerebral white matter lesions and depressive symptoms in elderly adults. Arch. Gen. Psychiatry.

[54] Mungas D., Harvey D., Reed B.R., Jagust W.J., DeCarli C., Beckett L., Mack W.J., Kramer J.H., Weiner M.W., Schuff N. (2005). Longitudinal volumetric MRI change and rate of cognitive decline. Neurology.

[55] Nordahl C.W., Ranganath C., Yonelinas A.P., Decarli C., Fletcher E., Jagust W.J. (2006). White matter changes compromise prefrontal cortex function in healthy elderly individuals. J. Cogn. Neurosci..

[56] Alonso A., Mosley T.H., Gottesman R.F., Catellier D., Sharrett A.R., Coresh J. (2009). Risk of dementia hospitalisation associated with cardiovascular risk factors in midlife and older age: The Atherosclerosis Risk in Communities (ARIC) study. J. Neurol. Neurosurg. Psychiatry.

[57] Englund E. (2002). Neuropathology of white matter lesions in vascular cognitive impairment. Cerebrovasc. Dis..

[58] Mascalchi M., Ginestroni A., Toschi N., Poggesi A., Cecchi P., Salvadori E., Tessa C., Cosottini M., De Stefano N., Pracucci G. (2014). The burden of microstructural damage modulates cortical activation in elderly subjects with MCI and leuko-araiosis. A DTI and fMRI study. Hum. Brain Mapp..

[59] Duering M., Finsterwalder S., Baykara E., Tuladhar A.M., Gesierich B., Konieczny M.J., Malik R., Franzmeier N., Ewers M., Jouvent E. (2018). Free water determines diffusion alterations and clinical status in cerebral small vessel disease. Alzheimer’s Dement..

[60] Maillard P., Fletcher E., Harvey D., Carmichael O., Reed B., Mungas D., DeCarli C. (2011). White matter hyperintensity penumbra. Stroke.

[61] Maillard P., Carmichael O., Harvey D., Fletcher E., Reed B., Mungas D., DeCarli C. (2013). FLAIR and diffusion MRI signals are independent predictors of white matter hyperintensities. AJNR Am. J. Neuroradiol..

[62] Promjunyakul N.O., Lahna D.L., Kaye J.A., Dodge H.H., Erten-Lyons D., Rooney W.D., Silbert L.C. (2016). Comparison of cerebral blood flow and structural penumbras in relation to white matter hyperintensities: A multi-modal magnetic resonance imaging study. J. Cereb. Blood Flow Metab..

[63] Ter Telgte A., van Leijsen E.M.C., Wiegertjes K., Klijn C.J.M., Tuladhar A.M., de Leeuw F.E. (2018). Cerebral small vessel disease: From a focal to a global perspective. Nat. Rev. Neurol..

[64] Duering M., Csanadi E., Gesierich B., Jouvent E., Herve D., Seiler S., Belaroussi B., Ropele S., Schmidt R., Chabriat H. (2013). Incident lacunes preferentially localize to the edge of white matter hyperintensities: Insights into the pathophysiology of cerebral small vessel disease. Brain.

[65] Jiaerken Y., Lian C., Huang P., Yu X., Zhang R., Wang S., Hong H., Luo X., Yap P.T., Shen D. (2021). Dilated perivascular space is related to reduced free-water in surrounding white matter among healthy adults and elderlies but not in patients with severe cerebral small vessel disease. J. Cereb. Blood Flow Metab..

